# A Novel Vehicle Tracking ID Switches Algorithm for Driving Recording Sensors

**DOI:** 10.3390/s20133638

**Published:** 2020-06-29

**Authors:** Yun Zhao, Xiang Zhou, Xing Xu, Zeyu Jiang, Fupeng Cheng, Jiahui Tang, Yuan Shen

**Affiliations:** 1 School of Information and Electronic Engineering; Zhejiang University of Science and Technology, Hangzhou 310023, China; yunzhao@zust.edu.cn (Y.Z.); 221801852131@zust.edu.cn (X.Z.); 221801852041@zust.edu.cn (Z.J.); 221801852017@zust.edu.cn (F.C.); 221801852086@zust.edu.cn (J.T.); 221801852079@zust.edu.cn (Y.S.); 2 School of Mechanical and Energy Engineering; Zhejiang University of Science and Technology, Hangzhou 310023, China

**Keywords:** YOLO, DDR, vehicle tracking, occlusion, ID switches

## Abstract

The main task for real-time vehicle tracking is establishing associations with objects in consecutive frames. After occlusion occurs between vehicles during the tracking process, the vehicle is given a new ID when it is tracked again. In this study, a novel method to track vehicles between video frames was constructed. This method was applied on driving recorder sensors. The neural network model was trained by YOLO v3 and the system collects video of vehicles on the road using a driving data recorder (DDR). We used the modified Deep SORT algorithm with a Kalman filter to predict the position of the vehicles and to calculate the Mahalanobis, cosine, and Euclidean distances. Appearance metrics were incorporated into the cosine distances. The experiments proved that our algorithm can effectively reduce the number of ID switches by 29.95% on the model trained on the BDD100K dataset, and it can reduce the number of ID switches by 32.16% on the model trained on the COCO dataset.

## 1. Introduction

With the assistance of driving data recorders (DDRs), vehicular accident rates have been reduced by 15–30% and related costs have also considerably decreased [1]. Increasing numbers of automobile manufacturers are using DDRs as a basic configuration in their vehicles. DDRs have the most common image sensors and usually have very high resolution. These sensors collect data that help the electronic control unit (ECU) to identify the road conditions ahead and analyze the association of the driver’s view and potential accidents [2,3]. They can also help to objectively record risky driving [4]. The collected data are sent to a neural network for scene understanding [5].

DDRs collaborate with other sensors to obtain advanced information about the road ahead. Dangerous driving intensity (DDI) and dangerous driving behavior (DDB) [6] are used as auxiliary evaluation indicators for advanced driver-assistance systems (ADAS). The ADAS can judge the dangerous driving behavior through the estimation of DDI and DDB. ADAS will also compute accident-risk index (ARI) [7] through various sensors data. The ARI can help ADAS decide whether it is safe. ADAS can also obtain the driver’s drowsiness through sensors inside the vehicle to avoid fatigue driving [8].

### 1.1. Detection Methods

For object detection, massive deep learning models have high detection accuracy and speed. Faster RCNN (Region-CNN) [9] is a practical two-stage algorithm: obtaining region proposals and classification. You only look once (YOLO) [10] is an effective one-stage algorithm that is able to process images at 30 frames per second (fps). The YOLOv3-608 has a mAP (mean Average Precision) of 57.9% on COCO (Common Objects in Context) test-dev. YOLO uses DarkNet to complete feature detection. YOLOv3 takes advantage of residual network (ResNet) [11] and has a better network architecture, named DarkNet-53. DarkNet-53 is composed of two types of convolution kernels: 3 × 3 and 1 × 1. It also has a similar structure shortcut connection to ResNet. Compared with the previous versions, it has lower BILLION FLOATING-POINT OPERATIONS PER SECOND (BFLOPs). However, it can run at 2× speed with approximative accuracy. YOLOv3 uses three different kinds of multi-scale feature maps to detect an object, whereas YOLOv2 uses a passthrough layer.

### 1.2. Tracking Methods

The kernel correlation filter (KCF) [12] is a suitable algorithm for tracking targets and has good efficiency for tracking various objects [13,14,15,16,17]. It is combined with the discriminative appearance model to form the dual correlation filter (DCF). Artificial potential field-elaborated resistance (APFE-RN) [18] is a new path planning and tracking technology in the field of autonomous driving. It plans the optimal path based on the local current comparison method (LCCM). A dynamic tracking model (DTM) [19] has been designed to give the covariance matrix of state noise to compensate for the shortcomings of the KCF algorithm. Feedback linearization [20] combined with a decision mechanism is also used to control the tracking process. Finally, on the basis of integrated probabilistic data association (IPDA), modified smoothing IPDA (MSIPDA) [21] improves the fixed-lag smooth data association to improve tracking performance.

An integrated deep neural network [22] can be combined with a deep convolutional neural network to extract information about the road ahead, use the road area and the hidden information of the road to predict the lateral position of vehicles, and assist in the continuous positioning and tracking of the vehicles by satellite. The improved support vector machine (SVM) classification model [23], making full use of color and histogram of oriented gradient (HOG) features, also has a good effect on tracking. A fast manifold regularized context-aware correlation tracking algorithm [24] constructed on correlation filters and digging local manifold structure information from different types of samples also produces a good tracking effect. The particle filter (PF) integrated with the interactive multi-model (IMM) algorithm [25] can improve the tracking performance under the Global Navigation Satellite System (GNSS). This improved method has smaller errors when the positioning accuracy is constantly changing.

The visual-appearance-level and spatial-temporal-level dictionary learning (VSDL) [26] method uses a visual appearance level dictionary and a spatiotemporal level dictionary to obtain the coding coefficients of each walking cycle, and judges the dictionary through the representation coefficient discriminant term to achieve tracking. The feature attention block [27] is designed to focus on different local areas, summarize the local areas, and integrate them into the neural network to achieve re-ID for people. Furthermore, the multi-level slice-based network (MSN) [28] has been proposed to extract local and global features through two branches and merge the two features to obtain unique features to realize pedestrian re-ID. The ResNet-50 backbone is combined with a structured loss function that contains two cosine parameters. This combination improves the representation of re-ID. An online clustering algorithm [29] has been used for facial ID and can recognize multiple targets at the same time. Finally, deep-person [30] is a new framework that achieves highly differentiated goals through a three-branch framework.

The accuracy of the KCF algorithm is 0.732. However, tracking multi-scale targets remains difficult. In KCF, the target box is set in advance and its size does not change during the tracking process. Therefore, in a tracking scene where the target size changes, KCF tracking is insufficient, and its recognition speed is considerably lower in complex recognition systems.

### 1.3. SORT and Deep SORT

Simple online and real-time tracking (SORT) [31] is an excellent and effective fast-tracking algorithm. SORT calculates the intersection over union (IOU) between two frames by detecting the frames at two consecutive moments, using the Hungarian algorithm for correlation. The 260 Hz speed of the SORT algorithm is many times faster than other similar tracking algorithms. However, the SORT algorithm pursues speed only and does not focus on the appearance of the target. The state of the target can be determined and associated using its position and each frame’s detection box. The SORT algorithm assigns an ID to each target that is identified and tracked successfully; however, when only considering its position and IOU, if the tracked target overlaps in continuous frames, the tracking effect of the SORT algorithm is considerably reduced. When the target appears again after overlapping it will be assigned a new ID, causing severe ID switches. Wojke [32] optimized the ID switches problem in the SORT algorithm and extracted the target features using a neural network. An eight-dimensional vector was used to represent the state of the target at a certain moment. For each tracking target’s motion trajectory, a threshold was set, and if the threshold was exceeded, the trajectory was considered as completely disappearing. After successfully tracking the target and generating the motion track, the state is set as a confirmed flag, and then the successfully tracked target is stored in the tracked storage.

According to the above literature review, vehicle tracking is important for counting the number of vehicles on the road and analyzing their trajectories. Accuracy is already high in the field of vehicle detection, but for vehicle tracking each target is associated in consecutive frames of the camera, and the same target is assigned the same ID for different frames. Some of the previous target tracking algorithms do not focus on the appearance characteristics of the target and some fail to track multi-scale targets.

The algorithm proposed in this study optimizes the ID switches that occur after being blocked for a long time. The algorithm calculates the Mahalanobis distance between the detection target and the prediction target, as well as the cosine distance fused with the appearance metric. The Euclidean distance is calculated and multiple thresholds are set for the Euclidean distance. The matching between the prediction target and the detection target is more accurate and the occurrence of ID switches is effectively reduced. Furthermore, in this experiment, we use Deep SORT as a starting point and mainly solve the tracking of vehicles in the video, effectively reducing the ID switches due to overlapping. A novel method to deal with ID switches is proposed. Due to the occurrence of occlusion in traffic, ID switches are common. By calculating the distance between the prediction box and the tracking box, a threshold relationship is established to reduce the number of ID switches.

## 2. Materials

### 2.1. Datasets

The MS COCO dataset [33,34,35] is a large target detection dataset introduced by Microsoft Corporation that has 80 categories and 330,000 pictures. It focuses on three points: target detection, contextual connection between targets, and localization of two-dimensional targets. It has 1.5 million object instances. Most of the pictures are from the real world, so the background is more complicated. Each image has an average of 7.7 instance targets. Additionally, there are more small goals.

BDD100K [36,37] is a diverse database for automatic driving, containing many videos collected by the Berkeley AI Research Team. The images extracted from these videos were applied as a training dataset, with 70,000 used for training, 20,000 for testing, and 10,000 for validation. It is a comprehensive dataset, including 10 types of targets: bus, light, sign, person, bike, truck, motor, car, train, and rider.

There are 100,000 images from videos in BDD100K. There is a total of 1.82 million ground truth data with 1.02 million including vehicles in BDD100K. The BDD100K dataset was used as a training dataset. It consists of 100,000 videos. The length of each video was 40 s and the resolution was 1280 × 720. The videos were collected by the Nexar application (Nexar, Tel Aviv-Yafo, Israel) and included GPS/IMU (Global Position System/ Inertial measurement unit) information. The entire dataset had 10 classes, mainly including targets on the road. The car category was trained in this research. 

**Figure 1:**
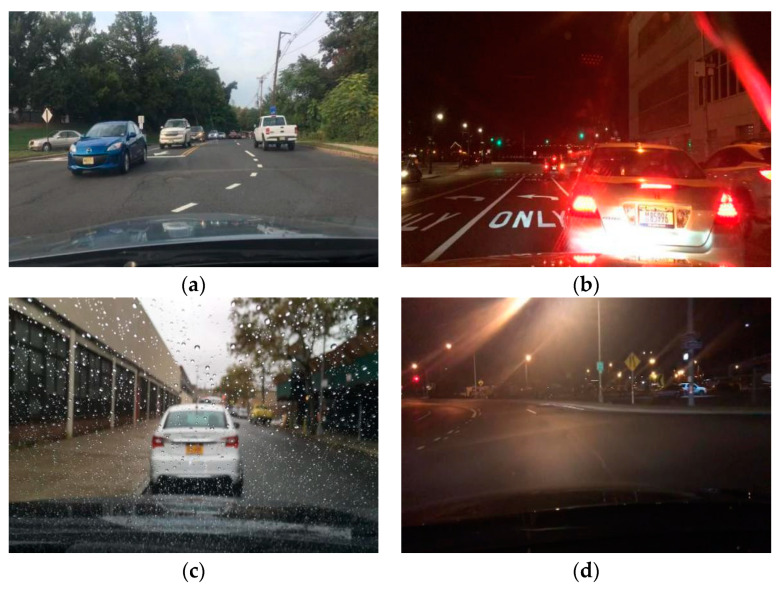
Examples of pictures of various weather and temporal conditions in the BDD100k dataset: (**a**) sunny weather; (**b**) night time; (**c**) rainy weather; and (**d**) foggy weather.

The results of training in complex weather and urban environments can effectively improve the applicability of the algorithm. This dataset is complex for a variety of reasons. The first is weather, with a variety of weather conditions presented, such as sun, rain, fog, and snow. The second is the time complexity as videos were captured during various time periods throughout the day and night. The third is the urban environment, characterized by various street environments. Figure 1 depicts examples of various weather/temporal conditions in the BDD100K dataset. 

### 2.2. Equipment

The proposed system was mainly used for vehicle detection and tracking. The processing unit was a Dell G3 3579 laptop, with an Intel^®^ Core™ i5-8300H CPU @ 2.3 GHZ, 8 G memory, a NVIDIA GTX 1060 graphics card with 6 G video memory, and the Windows 10 operating system. The model training was conducted on a desktop computer with an Intel^®^ Core™ i7-9700 CPU @ 3.0 GHZ processor, with 32 G memory, a NVIDIA GTX 2080Ti graphics card, video memory of 11 G, and the operating system Windows 10. The software system used pycharm2018 with Python 3.6.2, Tensorflow-gpu 1.5.0, Keras 2.1.4, and OpenCV library.

The camera model was a GM1910, the aperture was f/1.6, the exposure time was 1/50, the white balance was automatic, the ISO was 800, the focal length was 4.76 mm, and no flash was used. The frame rate of each video was 30.11 fps. The resolution of the video was 1920 × 1080. The camera configuration is shown in Table 1.

**Figure 2:**
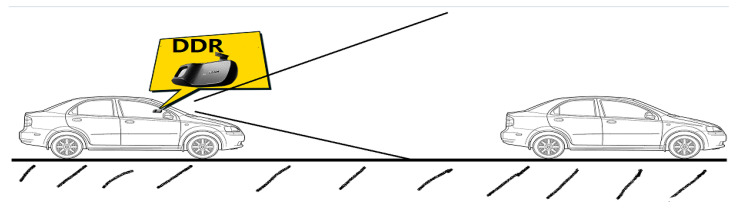
The structure of the proposed system: DDR (Driving Data Recorder) was placed in the vehicles.

The RGB camera sensor was placed in the vehicles’ holder and the shooting direction was the driving direction of the vehicles. The configuration of the video capture system is shown in Figure 2. The video obtained by DDRs is MJPG video. We extracted the frames from the acquired MJPG videos, and converted the RGB pictures to BGR pictures. The converted picture can be fed into the neural network to be recognized.

## 3. Methods

### 3.1. Detection Algorithm

The purpose of object detection is to detect objects in a single image captured by a single camera. The main purpose of object tracking is to establish a unified relationship between multiple photos. Multiple pictures are captured by multiple cameras or captured continuously by the same camera. Most object tracking tasks are re-identification. Re-identification emphasizes the detection of specific targets across cameras. Tracking requires a relatively short detection time, thus YOLO is the best choice for detection of the steps of object tracking. Since our experiment was based on the tracking of objects in a video, an algorithm for establishing a relationship between the same target in consecutive video frames to complete the tracking task was needed.

**Figure 3:**
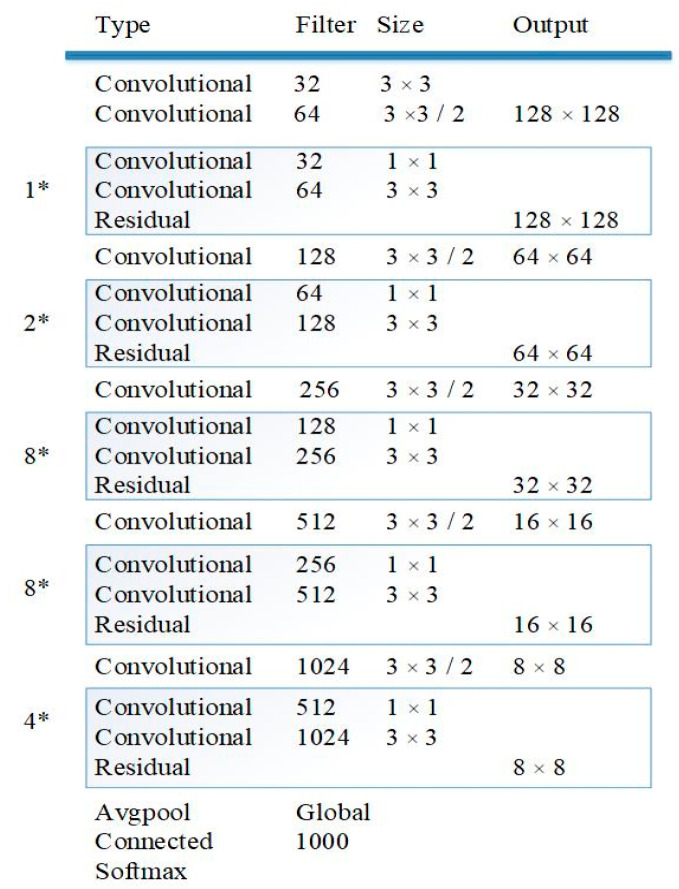
The architecture of the convolutional neural network.

The YOLO algorithm has a first-class detection speed among all the available object detection algorithms. In this experiment, because the algorithm needs to achieve real-time tracking, YOLO was selected for object detection. YOLO consists of 53 convolutional layers and each convolutional layer is followed by a layer of batch normalization (BN) and leaky rectified linear units (ReLU). YOLO has five different sizes of ReLU components. The architecture of the YOLO used in the experiment is shown in Figure 3.

The YOLO algorithm undergoes three feature concatenations. The detection layer finishes the detection at three different sized feature layers with three sized strides. The non-maximum suppression (NMS) algorithm finds the best object detection position by removing redundant (cross-repeated) boxes. In our experiment, we stored the detected objects in a list named *det* and the scores of the objects in a list named *sco*, and we fed *det* and *sco* into the NMS algorithm together. The objects detected by the YOLO algorithm were stored in a list, which were then used by the Kalman filter to predict its next position.

### 3.2. Tracking with Modified Deep SORT

The Modified Deep SORT optimizes the tracking stage ID switches. Compared with the Deep SORT algorithm, Modified Deep SORT proposes a new ID processing method. ID switches are very common due to blocking in traffic. By calculating the Euclidean distance between the detection box and the tracking box, and fusing the Euclidean distance with the Mahalanobis distance and the cosine distance, a threshold is also established to reduce the number of ID switches.

For each bounding box on YOLO’s detected result, the position information of all detected targets stored in an eight-dimensional matrix is extracted and used to represent the current state of the target. The matrix is given by

(1) $$p=\left(x,\;y,\;a,\;h,\;x^{\prime },\;y^{\prime },\;a^{\prime },\;h^{\prime }\right)\;$$

where (*x*,*y*) is the position of the bounding box, a is the aspect ratio of the bounding box, h is the height of the bounding box, and $(x^{\prime },y^{\prime },a^{\prime },h^{\prime }$) is the corresponding velocity information in the coordinate, which is (0,0,0,0) initially.

The moving track was divided into three states: (1) a tentative state, to be further observed; (2) a confirmed state, which has been matched successfully; and (3) a deleted state, which cannot be matched and should be deleted.

The state of the current target is fed into the Kalman filter, and the target is predicted and updated. The predicted result is stored in the tracked list. The result predicted by the Kalman filter is set to the tentative state and the matched state is set to the confirmed state, and the no longer appearing and cannot be matched results are set to the deleted state. The position information of the target set to the confirmed state is stored in the track matrix.

The Mahalanobis distance is used to calculate the distance between the predicted position and the detection position. As the tracking scene is a changing movement and tracking and detection are features of two different scales, it is reasonable to use Mahalanobis distance for correlation. Let the *b*th detection position match the *a*th prediction trajectory. In the Kalman filtering outcome, the covariance matrix of the *b*th predicted position is obtained, and then the prediction measurement of the *a*th position and the detection of the *b*th position are considered. With these three main parameters, the corresponding Mahalanobis distance is given by:

(2) $$dis_{m}\left(a,b\right)={\left(d_{b}-y_{a}\right)}^{T}S_{a}^{-1}\left(d_{b}-y_{a}\right)$$

where *a* is the order number of predicted position and *b* is the order number of detected positions. (*y_a_*,*s_a_*) is the measurement of the *a*th tracking, and *d_b_* is the measurement of the *b*th detection.

The use of Mahalanobis distance only considers the distance relationship between the detection target and the prediction target, so the surface characteristics of the target are ignored. Therefore, the appearance metric was introduced. We incorporated the appearance metric into the cosine distance and calculated the cosine distance containing the appearance metric. Combining the Mahalanobis distance with the minimum value of the cosine distance could improve the performance of the algorithm. The appearance metrics are obtained from a wide residual network. This network consists of two convolutional layers and six residual blocks. The features can be projected onto the unit hypersphere by the final batch and L2 normalization, so that it can be combined with the cosine metric. Then, we set Sb as the appearance metric descriptor and stored them, calculated the cosine distance between the order number of the *a*th tracking and the order number of the *b*th detection, and kept the minimum value of the cosine distances. The calculation of the cosine distance is given by

(3) $$dis_{c}\left(a,b\right)=min\left\{1-s_{b}^{T}s_{k}^{\left(a\right)}|s_{k}^{\left(a\right)}\in S_{i}\right\}$$

where *a* is the order number of predicted positions, *b* is the order number of detected positions, and *S_i_* contains each track’s appearance descriptors. For each calculation of the cosine distance, we kept the minimum value.

The Mahalanobis distance is good for short-term prediction and matching, but long-term occlusion causes tracking failure because it ignores surface features. It is important to incorporate an appearance metric into the cosine distance. For the previously calculated Mahalanobis and cosine distances, a fusion was required. The calculation of the fusion is given by

(4) $$U_{a,b}=vdis_{m}\left(a,b\right)+\left(1-v\right)dis_{c}\left(a,b\right)$$

where *dis_m_*(*a*,*b*) is the Mahalanobis distance of the ***a***th tracking and the ***b***th detection, *dis_c_*(*a,b*) is the cosine distance of the ***a***th tracking and the ***b***th detection, and *U_a,b_* is fused by the weight *v*. *v* is a hyperparameter used to adjust the weights of two items. It is usually set to 0.1.

We also applied a cascading matching strategy. Because a target that has been blocked for a long time may not even appear, its variance will increase. This cascade match strategy prioritizes closer targets.

The ID switches problem is serious in the field of vehicle tracking and needs to be improved. In a complex traffic environment, the mobility of the vehicles causes several occlusions per second. Each occlusion may cause a change in the tracking ID and even transmit one car’s ID to another car. After each occlusion, the next position of the vehicle is uncertain and the vehicle may not appear again. Using Deep SORT for vehicle tracking has higher requirements for handling overlaps because the recognized area of the vehicles is large and the surface of each vehicles may obstruct other vehicles. For vehicle occlusion on the road, the vehicles are relatively moving. The DDR shooting angle is from the inside of the moving vehicles, facing forward, so the probability of encountering occlusion is higher, and the vehicle in front could be blocked by other objects such as a vehicle or a traffic obstacle. After occlusion occurs, its ID will change when it is detected again.

Thus, a novel ID switches elimination algorithm is proposed in this paper with an improved tracking scheme that can reduce the number of ID switches. The collection of tracks is further arranged to remove part of the prediction results due to occlusion. In this scheme, the Kalman filter predicts that successful boxes will be stored in a new list. It sets time intervals to destroy tracking results in the list that have not been updated for a long time. The corresponding tracking ID is also stored in a new ID list. Starting from the second frame, a new tracking target may be generated due to a new detection target. For each newly generated tracking target, its Euclidean distance is calculated between it and the target saved in the tracking list previously. The smallest Euclidean distance is taken as the distance result. Because vehicles will not suddenly appear around the center of the video, for targets that do not appear around the boundary of the frame, the position of the newly predicted target is retained and the previous predicted position is deleted. In the vehicle tracking scene, the camera’s shooting angle is facing forward. Every tracked vehicle will enter the camera’s sensing area from one direction and leaves the sensing area from the other direction. Therefore, if the vehicle being tracked again after being overlapped in the perception area, it usually appears in the non-edge area of the picture. If it appears out of the edge of the screen, it will not be captured. Only targets that have never been tracked before will enter the picture from the edge area of the picture. Therefore, the position of the target needs to be updated without changing its ID. Then, the ID list must be updated, the ID of the new prediction target will be deleted, and the old ID will be used. These operations keep the old ID, but its predicted position is updated to the new one. The Euclidean distance is calculated by

(5) $$d_{o}=\min\left(\sqrt{{\left(p_{j}-p_{i}\right)}^{2}+{\left(q_{j}-q_{i}\right)}^{2}}\right)$$

where *o* is the Euclidean distance of the previous tracks and the current tracks, *p* is the *x* of the track box, *q* is the *y* of the track box, *j* represents all the prediction boxes in current frame, and *i* represents all the prediction boxes in the previous frames.

For the obtained Euclidean distance, a threshold value is used to complete the deletion of the box. In each frame, the distance between the prediction frame and the center of the frame is calculated as the evaluation index of the threshold, and the defined threshold is [10,100,300,550]. It is divided according to the size of the vehicle and the Euclidean distance in the image. Here, we need a threshold function defined as

(6) $$Z_{\left(a,b\right)}=\prod \left[d_{o}\left(a,b\right)\leq t^{\left(1\right)}\right]$$

where *Z*_(*a,b*)_ is the threshold distance, *d_o_* is the Euclidean distance of the previous tracks and the current tracks, and *t* is the list of thresholds.

The input of the modified Deep SORT algorithm is the continuous image frames captured by the camera. After the algorithm processes, the detected targets are bounded by bounding boxes, the objects in the continuous frames are associated, and the same target is assigned the same mark in each frame and give same target is assigned a uniform label. The output is the processed image frames. The description of this algorithm is shown in Algorithm 1.
**Algorithm 1:** Modified Deep SORT.YOLO detects the vehicles in the video frame and saves the position and ID of the detection boxes.Kalman filtering predicts the position of the vehicles while saving the position and the ID of the predicted boxes.In the subsequent detection and prediction process, all newly predicted frames are stored in a temporary unit.For each newly predicted frame position, position division is performed and different thresholds are set for different position areas. Compare with the positions of all predicted boxes that have appeared, and calculate the distance between the newly appearing box and the previously appearing boxes. When the Euclidean distance to a certain box that has appeared before is shorter than the set threshold, this is considered its next position.Update the position of the previously predicted box to the position just newly predicted and wipe the ID of the newly predicted box. Match the new predicted position with the old ID.

**Figure 4:**
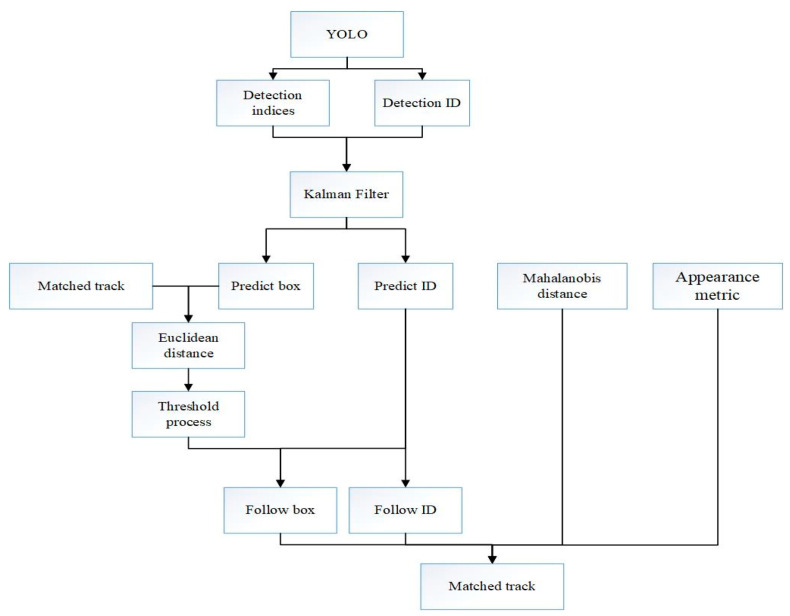
The flowchart of the recognition and tracking algorithm.

The flowchart of the recognition and tracking algorithm is shown in Figure 4. First, the image frame obtained by the camera is detected by YOLO. YOLO marks the location of the vehicles, stores the location information in the indices, and assigns a detection ID. Second, the Kalman filter predicts the next position of the detected target, stores the predicted result, and assigns a prediction ID. The Mahalanobis distance is calculated between the predicted and detected positions, and the cosine distance including the appearance metric is calculated. The Euclidean distance between the previously tracked target and the predicted target is also calculated. After threshold processing, the predicted target can be matched with the detected target.

### 3.3. Precision Performance Estimation

For the outcome trained by YOLO, the accuracy of the model was estimated using the average precision (AP) of vehicle detection based on different recalls. The true positive (TP) is the number of successfully identified vehicles in the vehicle category, the false positive (FP) is the number of objects identified as vehicles in the non-vehicle category. We calculated the TP and FP, as well as the AP, which is the average of the sum of all precisions. The accuracy of each category represents the recognition performance of the algorithm model on that category,

(7) $$Precision_{vehicle}=\frac{TP}{FP+TP}\;$$

where *Precision_vehicle_* is the average precision of the vehicle category, *TP* is the true positives in the vehicle category, and *FP* represents the false positives in the vehicle category.

### 3.4. Tracking Performance Estimation

For the tracking outcome, the number of ID switches was calculated. Due to occlusion or re-identification, the same vehicles can be assigned a new ID more than once during the continuous tracking process. Therefore, the number of ID switches was calculated as the main optimization goal for this novel algorithm. The total number of vehicles in consecutive video frames, the number of successful trackings, and the number of untracked objects were counted as auxiliary reference indicators.

## 4. Results

### 4.1. Detection Result

**Figure 5:**
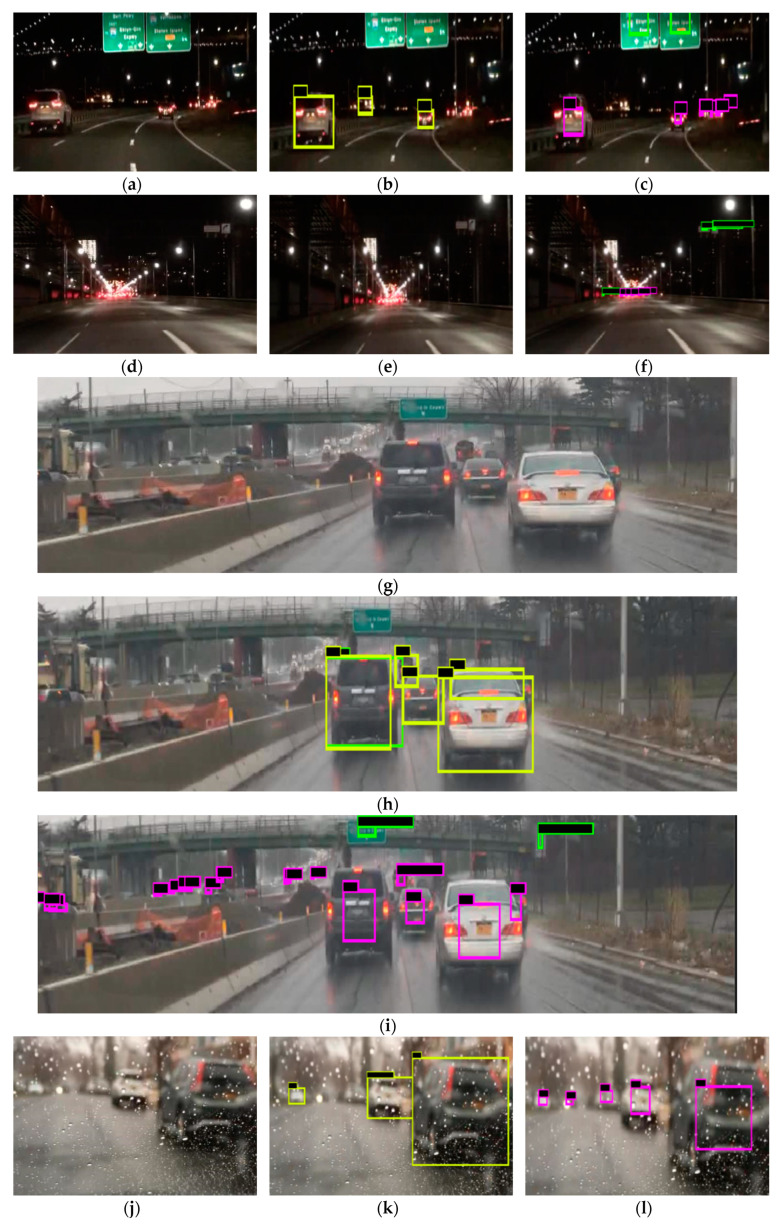

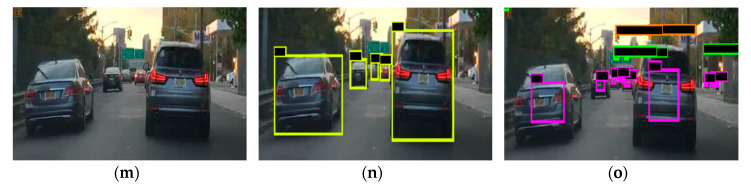
The figure (**a**–**o**) show the results of multiple pictures detected by models trained on two different datasets. Images in figure (**a**,**d**,**g**,**j**,**m**) are raw pictures. Images in figure (**b**,**e**,**h**,**k**,**n**) are detected by the model trained on COCO dataset. Images in figure (**c**,**f**,**i**,**l**,**o**) are detected by the model trained on BDD100K dataset. The same pictures used two models trained by two datasets to detect the object. The yellow boxes indicate the model with YOLO that was trained on the COCO dataset to detect vehicles. The purple boxes indicate the model with YOLO that was trained on the BDD100K dataset to detect vehicles. The green and orange boxes are the models trained by YOLO on the BDD100K dataset to detect traffic signs and traffic light categories, respectively.

Compared with the 55% average precision (AP) of car detection obtained from COCO dataset training, the AP of car detection on the BDD100K test dataset was 52%. When at night, rainy, or foggy and detecting distant vehicles, the performance of the BDD100K trained model is superior to the COCO trained model. The results of the detection are shown in Figure 5, which mainly compares the model training on the two datasets for vehicles detection. In Figure 5, the yellow boxes are the detection results for the YOLO algorithm trained on the COCO dataset, and the purple boxes are the detection results for the YOLO algorithm trained on the BDD100K dataset. The images in Figure 5a–f were acquired at night. The model detected more vehicles in Figure 5c than in Figure 5b, and had a higher accuracy rate for small and distant targets in Figure 5f than in Figure 5e. On rainy or foggy days, the model trained on the BDD100K was able to effectively recognize occluded targets in Figure 5i compared with Figure 5h. On sunny days, the model trained on the BDD100K had more accurate recognition performance for small targets in Figure 5o than in Figure 5n.

### 4.2. Tracking Results

For the tracking performance, the main indexes that were observed included the number of vehicles successfully identified and tracked in the video. The number of ID switches is an important index in the tracking phase.

The experimental results were judged by the following metrics:

(1) Secs: Seconds of each video.
(2) Numbers: Number of vehicles in each video.
(3) Frames: Number of frames in each video.
(4) Boxes: Number of detection boxes in each video
(5) FPs: Number of the false detected vehicles in each video.
(6) FNs: Number of missed detected vehicles in each video.
(7) MTs: Number of the mostly tracked vehicles during its presence in each video.
(8) MLs: Number of the mostly lost vehicles during its presence in each video.
(9) ID switches: Times of the ground-truth tracks’ identity changes.

Table 2 provide comparations of the detection and tracking performance between our improved algorithm and the original algorithm after training on the BDD100K dataset. Rows 2–9 are all in the daytime scene. Rows 10–17 are all in the night scene. Every two rows are the same video used in two methods.

Table 3 provides comparations of the detection and tracking performance between our improved algorithm and the original algorithm after training on the COCO dataset. Rows 2–9 are all in the daytime scene. Rows 10–17 are all in the night scene. Every two rows are the same video used in two methods.

The above tables list the results of vehicle tracking in multiple videos. In the experiments, the improved and the original algorithms were used to compare the effects of tracking. As shown in the tables, the MDS algorithm can effectively reduce ID switches. In each sample video, ID switches are reduced by different amounts. The reasons for these differences include the number of vehicles in the sample videos and the number of occlusions. In Rows 14 and 15 of Table 3, the number of ID switches only dropped by one because there are fewer vehicles in this sample video, as well as less occlusion between vehicles during tracking. When the target is blocked for a long time, the vehicle will move a larger distance in the frame, and it is hard to associate with the position before occlusion so ID switches are only reduced by one. Therefore, the important attribute that affects ID switches is occlusion, and the important attribute that affects occlusion is occlusion time.

After the above experiments, the proposed algorithm was found to effectively detect and track vehicles on the road, and can effectively reduce the occurrence of ID switches. The model trained on the BDD100K dataset was able to track the target for a longer time and have a longer life span. Therefore, the model trained by BDD100K produced a larger number of detections. Comprehensive analysis of the above eight videos shows 29.95% and 32.16% reductions in ID switches on the model trained on the BDD100K and COCO datasets, respectively.

For tracking speed, the modified algorithm processes pre-shot videos at 4.5 fps. In the case of real-time processing using a camera, the speed is 6.5 fps.

## 5. Discussion

In this experiment, the video capture sensor was a driving data recorder. In traffic, DDRs are common as they can sense and record road information. DDRs use a forward-looking perspective to collect information about the road ahead, thus the vehicles generate a lot of occlusion. The method proposed in this experiment can detect vehicles in the video frames collected by DDRs and can achieve the real-time tracking of vehicles by predicting the recognition target.

In the experiment of detection, we found that the recognition of the model trained on the COCO dataset was more precise and better covered the entire target. However, the model trained on the BDD100K dataset performed better at night and in rain, and provided advantages for small target detection. Due to the better detection performance produced by training on the BDD100K dataset, the tracking process has a longer life span and the recognition of most occluded objects was excellent. The model trained on the BDD100K dataset can better detect small targets during the detection phase, thereby reducing most of the objects lost during the tracking phase. However, it produces a certain number of errors, such as detecting the wheel of a truck as a car. Therefore, the scope of the dataset needs to be expanded to further train the model.

During the tracking phase, the proposed MDS algorithm reduced ID switches more stably at night. The MDS algorithm can markedly decrease the number of ID switches, but a certain number of ID switches still occur. To solve this issue, more attention should be paid to appearance features, and the recognition speed can also be improved. In the process of DDR video shooting, a higher-resolution camera can be used to provide better photos for recognition, which would help to improve the recognition effect.

Because the weather is clear, partly cloudy, overcast, rainy, snowy, and foggy in the BDD100K dataset, and the videos were captured at dawn, dusk, daytime, and nighttime, the dataset has more training pictures at night and in rain. Therefore, in the vehicle detection stage, the model trained on the BDD100K dataset performs better at night and in rain than the model trained on the COCO dataset. During vehicle tracking in the video samples, the detection effect at night and in rain was stable. Some vehicles were hard to recognize for the model. Because some vehicles were not detected by the model during the entire cycle captured by the camera, they were not detected again after being occluded, which reduced the chance of ID switches and provided more stable performance.

## 6. Conclusions

In this study, the performances of the Deep SORT and modified Deep SORT algorithms were compared on the BDD100K and COCO datasets. The videos were captured by a DDR and used to detect and track targets using the algorithms in this experiment. The findings proved that the MDS algorithm can track vehicles based on recognition and effectively reduce the generation of ID switches. The models trained on the BDD100K and COCO datasets all performed well in terms of detection. For the model trained on the COCO dataset, the ID switches were reduced by 32.16% when tracking, and by 29.95% for the model trained on the BDD100K dataset. This new algorithm applied to DDR fully proves its effectiveness. This DDR algorithm would be beneficial for vehicle tracking in driving recorded videos. In future work, we will focus on tracking vehicles from different video cameras and proposing a better method to match the appearance features.

## Figures and Tables

**Table 1:** The configuration of the camera.

Camera Model	Aperture	Exposure Time	White Balance	ISO	Focal Length	Flash	FPS
GM1910	f/1.6	1/50	Auto	800	4.76 mm	No	30.11

**Table 2:** Samples of vehicle tracking using the MDS (Modified Deep SORT) and DS (Deep SORT) algorithms with a detection model trained on the BDD100K dataset.

Time	Method	Sec	Frames	Boxes	Num	FPs	FNs	MTs	MLs	ID Switches
daytime	BDD+DS	36	1110	5163	48	7	5	41	7	**55**
	BDD+MDS	36	1110	5163	48	7	5	41	7	**31↓**
	BDD+DS	33	1012	5831	30	2	9	21	12	**38**
	BDD+MDS	33	1012	5831	30	2	9	21	12	**27↓**
	BDD+DS	59	1794	12,270	92	11	2	48	11	**217**
	BDD+MDS	59	1794	12,270	92	11	2	48	11	**138↓**
	BDD+DS	31	941	8699	23	0	4	19	4	**38**
	BDD+MDS	31	941	8699	23	0	4	19	4	**27↓**
night	BDD+DS	57	1729	9091	68	7	0	59	9	**68**
	BDD+MDS	57	1729	9091	68	7	0	59	9	**56↓**
	BDD+DS	56	1684	7410	60	6	19	38	22	**80**
	BDD+MDS	56	1684	7410	60	6	19	38	22	**65↓**
	BDD+DS	31	929	2016	11	3	1	9	2	**17**
	BDD+MDS	31	929	2016	11	3	1	9	2	**13↓**
	BDD+DS	60	1831	16,486	46	0	3	40	6	**155**
	BDD+MDS	60	1831	16,486	46	0	3	40	6	**90↓**

**Table 3:** Samples of vehicle tracking using the MDS and DS algorithms with a detection model trained on the COCO dataset.

Time	Method	Sec	Frames	Boxes	Num	FPs	FNs	MTs	MLs	ID Switches
daytime	COCO+DS	36	1110	4843	48	8	7	41	7	**52**
	COCO+MDS	36	1110	4843	48	8	7	41	7	**28↓**
	COCO+DS	33	1012	5312	30	9	4	26	4	**37**
	COCO+MDS	33	1012	5312	30	9	4	26	4	**27↓**
	COCO+DS	59	1794	11,078	92	14	2	57	2	**305**
	COCO+MDS	59	1794	11,078	92	14	2	57	2	**229↓**
	COCO+DS	31	941	9629	23	0	6	17	6	**95**
	COCO+MDS	31	941	9629	23	0	6	17	6	**67↓**
night	COCO+DS	57	1729	7638	68	11	4	64	4	**62**
	COCO+MDS	57	1729	7638	68	11	4	64	4	**28↓**
	COCO+DS	56	1684	6064	60	5	9	50	10	**90**
	COCO+MDS	56	1684	6064	60	5	9	50	10	**75↓**
	COCO+DS	31	929	1315	11	4	1	8	3	**14**
	COCO+MDS	31	929	1315	11	4	1	8	3	**13↓**
	COCO+DS	60	1831	12,549	46	7	5	41	5	**90**
	COCO+MDS	60	1831	12,549	46	7	5	41	5	**44↓**
